# Access to, use, knowledge, and preferences for information technology and technical equipment among people with chronic obstructive pulmonary disease (COPD) in Sweden. A cross-sectional survey study

**DOI:** 10.1186/s12911-021-01544-4

**Published:** 2021-06-10

**Authors:** Pernilla Sönnerfors, Kirsti Skavberg Roaldsen, Agneta Ståhle, Karin Wadell, Alexandra Halvarsson

**Affiliations:** 1 Department of Neurobiology, Caring Sciences and Society, Division of Physiotherapy, Karolinska Institutet, Huddinge, Sweden; 2 Women’s Health and Allied Health Professionals Theme, Medical Unit Occupational Therapy and Physiotherapy, Karolinska University Hospital, Stockholm, Sweden; 3 Faculty of Health Sciences, UiT The Arctic University of Norway, Tromsø, Norway; 4 Department of Research, Sunnaas Rehabilitation Hospital, Nesodden, Norway; 5 Department of Community Medicine and Rehabilitation, Physiotherapy, Umeå University, Umeå, Sweden

## Abstract

**Background:**

The use of information technology can make pulmonary rehabilitation interventions in people with chronic obstructive pulmonary disease (COPD) more flexible and thereby has the potential to reach a larger proportion of the population. However, the success of using information technology in pulmonary rehabilitation is dependent on the end-user’s competence in information technology and access to the Internet. The aim was to describe the access to, and the use, knowledge, and preferences of information technology and technical equipment among people with COPD.

**Methods:**

Telephone interviews were conducted using a standardised questionnaire on information technology and technical devises addressing the household, access to and usage of the Internet, contact with authorities, e-commerce, security, the workplace, digital competence, and disabilities. Questions were also posed regarding participants’ views on a future eHealth tool for COPD, appropriate content, and the potential likelihood for them to use an eHealth tool for exercise training.

**Results:**

In total 137 persons agreed to participate, 17 dropped out resulting in 120 included participants (response rate 88%). The participants (86 women) were aged 51 to 92 years (mean: 72.5), and all severity grades of COPD according to GOLD A-D were represented. Over 90% had access to the Internet. Smartphones were used by 81%, and over 90% used apps. Participants had high knowledge of how to use the Internet, 91% had used the Internet during the last 3 months, 85% almost every day. The most common requests for a future eHealth tool for COPD were evidence-based and trustworthy information on COPD, (including medication, exercise training, inhalation and breathing techniques), communication (chat) with others and with health carers. Access to individually adjusted exercise training, and support, (motivation via prompts, chat rooms, digital information board) was also desired.

**Conclusions:**

The present study showed that people with COPD in Sweden have high access and ability to use the Internet and information technology. They are frequent users and most of them take part in the digital society, even to a higher extent than the general population. The results show that the use of an eHealth tool could be a suitable strategy for people with COPD.

## Background

Chronic Obstructive Pulmonary Disease (COPD) is a common disease affecting the whole body, but most prominently the lungs. COPD is most commonly caused by tobacco smoking or environmental exposure. The breathing difficulties are mainly caused by an airway obstruction that originates from permanent damage to the lung tissue [1]. The disease is one of the leading causes of mortality worldwide, and it is predicted to be the third leading cause of death in the 2020s. According to WHO 2017, 251 million people in the world were affected by COPD. Globally it is estimated that more than three million people die each year from COPD; i.e. 6% of all deaths worldwide [2]. Still the disease is largely underdiagnosed [3–5] and the prevalence differs depending on which definition/guideline criteria is used [3, 4, 6, 7]. The prevalence is higher among women than men in developed countries [7]. In Sweden approximately 500,000 people are affected, nearly 3000 of whom die annually due to the disease, and the mortality is increasing [8]. The severity of the disease can be divided in stages I–IV (I: mild–IV: very severe, reflecting airflow limitation according to a lung function test with spirometry) or in grades A–D (reflecting the burden of the disease with regard to symptoms and risk of exacerbation) [1]. A mild stage is most common [3]. During the last years, the prevalence of moderate to severe COPD has decreased in Sweden [9]. Comorbidities are common at all stages and make the picture of the disease more complex. COPD is associated with a high economic burden on society, on the affected people and their families, as well as on their daily life in general [1].

Pulmonary rehabilitation (PR) is recommended as standard care for people with COPD. It is described as a comprehensive intervention which is based on a full patient evaluation, followed by individualised treatment. The patient-tailored treatment often includes exercise training, education, and behavioural change strategies, aiming to improve the physical and emotional situation as well as the long-term adherence to health-enhancing behaviours [10]. Participating in PR has shown to be effective regarding quality of life, emotional state, physical capacity, and ability to participate in everyday activities and recently also decreased mortality [1, 10, 11]. Despite the known positive effects of PR, only a minority of the COPD population has access to this treatment [12].

The digitalisation is a rapid process worldwide and today, nine out of ten people use the Internet basically every day in Sweden. The pandemic of COVID-19 has put digital tools and the possibilities of the Internet to the test, but it has been shown that many Internet users have started using digital services more frequently than before or tested them for the first time during this particularly challenging time [13, 14]. Electronic health (eHealth) is described as the use of information and communication technologies (ICT) for improving health [15], where health-improving intervention is delivered electronically. Telerehabilitation includes “the use of ICT to provide rehabilitation services from a distance” and can involve different types of eHealth tools [16]. An eHealth tool is the way the content of information is comprised, i.e., presented, delivered, and received, by the program used (software). The eHealth tools used in clinical settings and in research studies vary in features and there is no consensus on which feature has the greatest impact on the outcomes. The technical equipment (device) used to deliver and receive information can also differ e.g.: computer, smartphone, tablet, and video-conference system. There is still uncertainty regarding access to and knowledge of information technology (IT) and technical equipment in the COPD population, which is important knowledge when designing, developing, or choosing among existing eHealth tools. The use of familiar technical equipment has been shown to be an advantage to reach higher exercise training compliance at a distance [17].

Using modern ways of communication and technical equipment can bring more flexible solutions to people with COPD and reach a larger proportion of the population [18]. Some recent studies on COPD and telerehabilitation have shown a decline in costs due to a reduction in staff, travel, and time benefits, which is important as health care costs for COPD are considerable [19–21]. Telerehabilitation shows similar results for exercise capacity as usual rehabilitation, when including the same exercise training program [22]. Perceptions and experiences (usability, adherence rates) of using eHealth tools for exercise training have shown to be positive among participants in general, while health care staff have been more sceptical [23]. Even the most severely impaired patients with COPD have been found to manage using an eHealth tool, adhere to a physical training program and therefor also benefit from PR [24]. Physical training distributed in a more accessible way, such as in customised eHealth tools, may improve the accessibility to and usability of physical activity and exercise training.

Statistics Sweden (ISO 20,252:2012 certified) is responsible for and coordinates the system for the official and government statistics in Sweden. The results are public and can be reached on the Eurostat website [25], in the Swedish statistical databases, and via social media. Statistics Sweden’s survey on IT and technical devises in households, has been carried out with a similar theme for 20 years. The quality of the survey has increased over the years, due to improved collection and selection processes (2012) and covering an expanded age span (2013) [14].

There is a lack of knowledge on a number of topics of IT, including access to and the use of, knowledge, and preferences in people with COPD. The present study was conducted in a Swedish COPD population using Statistics Sweden’s existing survey appropriate for the aim of this study [14].

The aim of the present study was to describe the access to and use, knowledge and preferences of information technology and technical equipment among people with COPD.

## Methodology

### Study design

This is a cross-sectional study with telephone interviews. The interviews included Statistics Sweden’s 2018 questionnaire on ICT usage in households and by individuals [14]. Ahead of the data collection, a pre-study was performed on a focus group with the purpose of discussing and adapting the questionnaire for this specific population. The STROBE guidelines [26] were used to report this study.

### Participants

The recruitment aimed to reach participants from different parts of Sweden by approaching health care providers and the national patient organisation representing different health care settings (hospital, rehabilitation centres, primary care setting) from all six health care regions in Sweden. The partakers received information and relevant materials by post or e-mail, including instructions to both recruiters and participants. A reminder was sent to all non-responding health care providers by post after 4–6 weeks.

The inclusion criteria for the patients were: a diagnosis of COPD, be aged more than 40 years, and able to understand the Swedish language sufficiently to answer the questions. The criteria were verified by the health care providers and by the interviewers when recruiting and contacting them. Participants were excluded if no written informed consent was returned. In the present study we aimed to reach the same representation in the Swedish COPD population as Statistics Sweden accomplished in their survey in the general population, and with the same response rate (45%). This meant recruiting 136 people with COPD to take part in the study.

### Questionnaire

Permission to use Statistics Sweden’s standardised questionnaire was obtained prior to start of the study. The questionnaire included more than 130 questions, addressing nine different areas; the household, access to the Internet, usage of the Internet, contact with authorities and public sector via the Internet, Internet commerce (e-commerce), security and the Internet, computers and the Internet in the workplace, digital competence, and disabilities [14].

Additional information on background and questions considering participants own views on a future digital tool, i.e., appropriate content, potential likelihood of their use of the tool suitable for exercise training, and questions concerning health and COPD (46 questions) were also posed. The COPD Assessment Test (CAT) [27], with questions on disease impact, and the Frändin–Grimby scale [28], assessing physical activity level, were also used and complemented with specific questions internally developed on comorbidities, living situation, smoking habits, etc. The Karolinska Institutet web survey platform, “KI Survey” by Sunet, Artologik’s survey and reports [29], was used for all data collection. The data was collected through telephone interviews from October 2019 to June 2020 and carried out by three highly experienced specialised physiotherapists, with more than 20 clinical work years at Karolinska University Hospital. To ensure reliable results, the three physiotherapists conducting the interviews communicated prior to the start and weekly during the study period. The time required for each interview varied between 20 and 60 min due to some answers leading to supplementary questions, and feedback given on topics raised by the participants.

### Pre-study

A pre-study was conducted before the full-scale study with participants with COPD, representing the end-users, participating in a focus group discussion. Two researchers were present, one who conducted the interview (PS) and one observer (KSR), together with three participants (1 woman and 2 men, aged: 68–74 years, severe to very severe COPD, part time working or not working), with “low” to “high” competence in IT and the use of technical equipment. One of the men participated by phone. All of them discussed any uncertainties concerning recruitment and content of Statics Sweden’s questionnaire, and questions concerning health and COPD. The material used in the focus group (questionnaire, instructions) was sent ahead to the participants. The focus group discussion required approximately two and a half hours and was audio recorded. Written summaries were made by both the moderator and the observer. Discussions on these summaries, written notes from the observing researcher and the audio recording were used in the adaptation of the phrasing of the written information, i.e., brochures for recruitment, introduction texts. The pre-study also resulted in five additional questions used in the survey: grading of level of knowledge and familiarity with the use of IT and technical equipment, use of Internet-based support for COPD, the content, function and prospects in a future eHealth tool, and the degree of probability of using a future eHealth tool designed for promoting physical activity and exercise training in COPD. The results from these questions were used to find participants’ preferences for a future eHealth tool.

### Statistics

Variables are presented as number (n), percentage (%), mean, standard deviation (SD), minimum and maximum. The statistical program SPSS (IBM SPSS Statistics 27, IBM, New York, US) was used to perform the statistical analysis. The data for age, gender and BMI were tested for normal distribution using the Kolmogorov–Smirnov normality test. The grouping used was formed according to the grouping used by Statistic Sweden (age, gender). Any missing data were handled according to described, recommended methods.

## Results

**Fig. 1:**
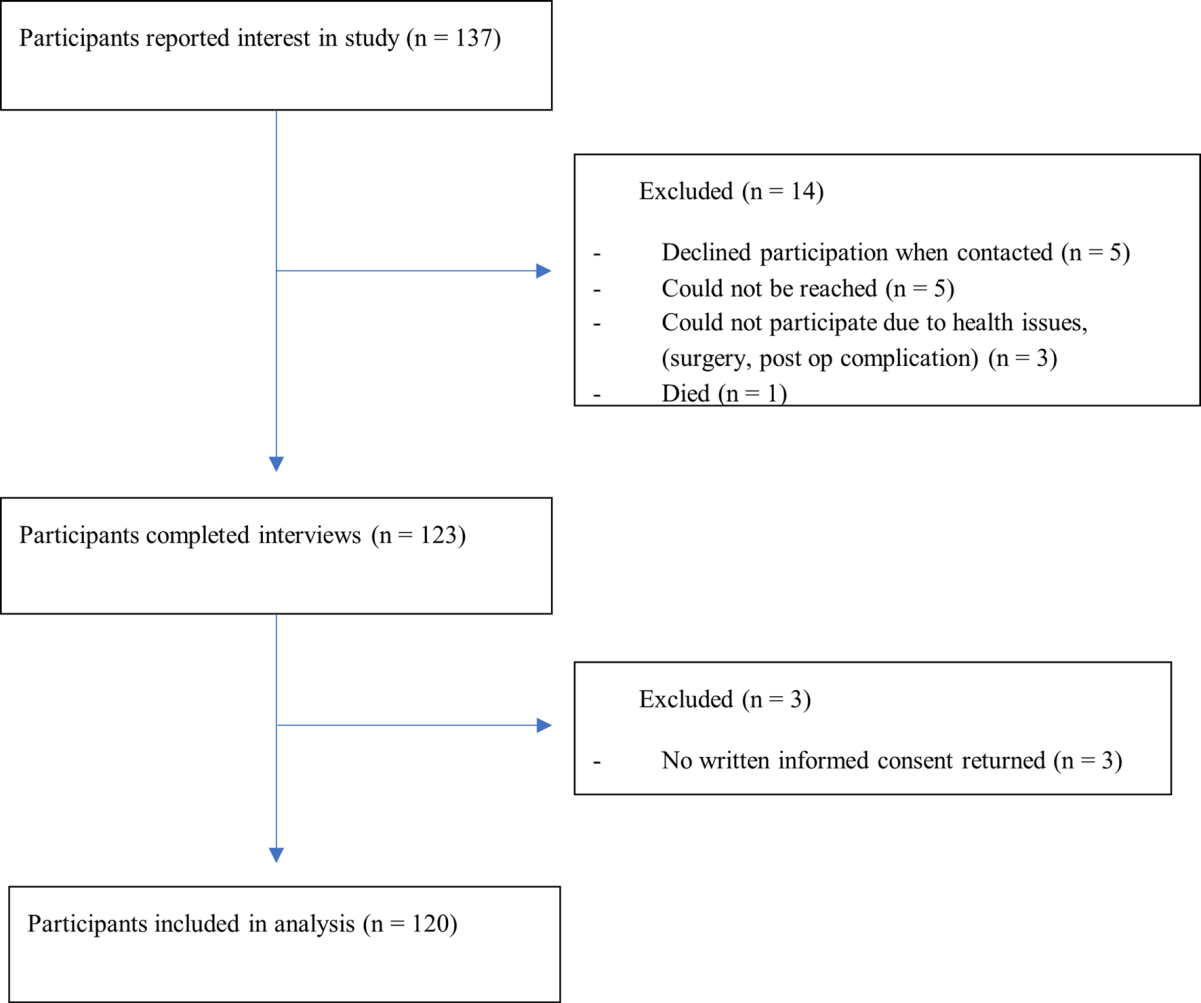
Flow chart for the inclusion of participants in a cross-sectional study on the Internet and technical equipment in people with chronic obstructive pulmonary disease

In total 137 people from all health care regions in Sweden participated in the present study with 17 dropouts (10 men, 7 women). Figure 1 shows the inclusion flow chart. Twenty-five of more than 100 contacted health care providers responded positively to help with recruitment, as did the national patient organisation.

A total of 120 participants were included (86 women, 34 men) aged 51 to 92 (mean: 72.5) years.

All severity grades of COPD according to the Global Initiative for Chronic Obstructive Lung Disease (GOLD) A-D were represented. According to the total CAT score, most of the participants had moderate disease impact due to COPD. More than half of the participants had one or more comorbidities. Detailed information about sociodemographic and disease-related data are presented in Table 1.

**Table 1:** The sociodemographic characteristics and disease-related data of the participants in the present study

	Total (n = 120)	Women (n = 86)	Men (n = 34)
*Gender, n (%)*			
Female	86 (72)		
Male	34 (28)		
*Age, mean, (SD),*			
Years	72 (7.7)	72 (7.8)	74 (7.6)
*Disease classification, n (%)*			
GOLD group A	21 (18)	18 (21)	3 (9)
GOLD group B	50 (42)	32 (37)	18 (53)
GOLD group C	5 (4)	1 (1)	4 (12)
GOLD group D	44 (37)	35 (41)	9 (26)
*Civil status, n (%)*			
Living alone	61 (51)	51 (59)	10 (29)
Living together	59 (49)	35 (41)	24 (71)
*Living condition, n (%)*			
Villa or house	34 (28)	22 (26)	12 (35)
Apartment	86 (72)	64 (74)	22 (65)
Domestic home care, n (%)	13 (11)	10 (12)	3 (9)
*Occupation, n (%)*			
Working	16 (13)	10 (12)	6 (18)
Not working	104 (87)	76 (88)	28 (82)
*Education status, n (%)*			
≤ 8 years	19 (16)	12 (14)	7 (21)
9 years	17 (14)	14 (16)	3 (9)
10–11 years	17 (14)	14 (16)	3 (9)
≥ 12 years	67 (56)	46 (54)	21 (62)
Oxygen treatment^a^, n (%)	10 (8)	7 (8)	3 (9)
α-1-antiprypsin deficiency, n (%)	3 (2)	2 (2)	1 (3)
*Smoking status, n (%)*			
Current smoker	16 (13)	11 (13)	5 (15)
Former smoker	100 (83)	73 (85)	27 (79)
Never smoker	4 (3)	2 (2)	2 (6)
BMI, mean (SD)	27 (5.5)*	27 (5.6)*	26 (5.0)
CAT total score, median	15	15.5	14.5
(min–max)	(1–36)	(2–36)	(1–31)
Frändin-Grimby activity scale^b^, median	4	4	3
(min–max)	(1–6)	(1–5)	(1–6)
Walking aid^c^, n (%)	39 (32)	33 (38)	6 (18)
*Comorbidities, n (%)*			
Hypertension	70 (58)	50 (58)	20 (59)
Heart disease^d^	38 (32)	26 (30)	12 (35)
Anxiety, depression	33 (28)	31 (36)	2 (6)
Osteoporosis	33 (28)*	30 (35)*	3 (9)
Cancer	26 (22)**	20 (24)*	6 (18)*
Diabetes	17 (14)	9 (11)	8 (24)
Sleep apnea syndrome	15 (12)	11 (13)	4 (12)
Stroke	8 (7)	5 (6)	3 (9)

BMI, Body mass index; CAT, COPD Assessment Test; COPD, Chronic obstructive pulmonary disease; GOLD group, Global Initiative for Chronic Obstructive Lung Disease group; HRQL, health related quality of life

^a^Oxygen treatment ≥ 16 h/d and/or at activity, ^b^A six-degree activity scale, including household activities, ranging from 1- “hardly any physical activity”, 2- “mostly sedentary”, 3- “easier physical exercise 2–4 h per week”, 4- “more strenuous exercise 1–2 h per week” 5- “more strenuous exercise > 3 h per week” to 6- “hard exercise several times a week”. ^c^Walking aid used outdoor walker, cane or other. ^d^Heart disease: myocardial infraction, heart failure, angina pectoris

*One missing value, ** two missing values

Over 90% of the participants had access to the Internet. Seven out of ten had access to a desktop Internet connection and two thirds to a mobile Internet connection. Smartphones were used by approximately 80% and over 90% used applications (apps) on their smartphones. For more detailed information about Internet access, see Table 2.

**Table 2:** Access to, and usage of the Internet among the 120 participants in the present study

	Total	Women	Men
	n = 120	n = 86	n = 34
*Access to Internet, n (%)*			
Yes	110 (92)	79 (92)	31 (91)
No	10 (8)	7 (8)	3 (9)
*Access to stationary connection, n (%)*			
Yes	84 (70)	59 (69)	25 (74)
No	25 (21)	19 (22)	6 (18)
Don’t know	11 (9)	8 (9)	3 (9)
*Access to mobile connection, n (%)*			
Yes	74 (62)	54 (63)	20 (59)
No	29 (24)	18 (21)	11 (32)
Don’t know	17 (14)	14 (16)	3 (9)
*Used internet, n (%)*			
During the last 3 months	108 (91)*	77 (91)*	31 (91)
> 3 months to 1 year ago	3 (2)	2 (2)	1 (3)
> 1 year ago	5 (4)	4 (5)	1 (3)
Never used	3 (2)	2 (2)	1 (3)
	n = 108	n = 77	n = 31
*Frequency of internet usage (last 3 months), n (%)*			
Almost every day	92 (85)	65 (84)	27 (87)
> once a week	12 (11)	8 (10)	4 (13)
< once a week	4 (4)	4 (5)	0 (0)
*Used internet several times/day (last 3 months), n (%)*			
Yes	89 (82)	62 (80)	27 (87)
No	18 (17)	15 (20)	3 (10)
Don’t know	1 (1)	0 (0)	1 (3)
*Use of technical devices (last 3 months), n (%)*			
Desktop computer	42 (39)	25 (32)	17 (40)
Laptop	74 (68)	52 (68)	22 (71)
Tablet	50 (46)	35 (46)	15 (48)
Cellphone or smartphone	96 (89)	71 (92)	25 (81)
Other mobile device^a^	5 (5)	5 (6)	0 (0)
	n = 111	n = 79	n = 32
Use of smartphone (ever), n (%)	90 (81)	67 (85)	23 (72)
Use applications on smartphone	85 (94)	65 (97)	20 (87)
*Activities performed on the Internet, (ever) n (%)*			
Sent or received email	101 (91)	72 (91)	29 (91)
Had phone call, video call^b^	57 (51)	42 (53)	15 (47)
Took part in social networking sites^c^	74 (67)	56 (71)	18 (56)
Searched for information (goods or services)	100 (90)	73 (92)	27 (84)
Searched for information (health related)	90 (81)	65 (82)	25 (78)
Booked an appointment^d^ (health care)	32 (29)	18 (23)	14 (44)
Financial activities	97 (87)	68 (70)	29 (30)
Courses	12 (11)	6 (8)	6 (19)

^a^Other mobile device: gaming console, reading tablet, smartwatch. ^b^Had phone, video calls via internet, Skype, WhatsApp, Facetime. ^c^Took part in social networking sites, Facebook, Twitter, Snapchat, Instagram. ^d^ Booked an appointment via a website with a physician, dentist, hospital, or health center

*One refused to answer

The majority of the participants had used the Internet during the last three months, and most of them used the Internet almost every day, several times a day. The most frequently used equipment for connecting to the Internet was (in descending order): mobile phone or smartphone, laptop, tablet, and a desktop computer. The Internet was mostly used for (in descending order): sending and receiving e-mails, searching for information about goods or services, financial activities, and home banking, searching for health-related information, participating in social networking sites, phone, or video calls via the Internet and for booking appointments with health carers. Detailed information about the usage of the Internet is presented in Table 2.

Participants had provided authorities and the public sector with information during contact via their websites or apps (73%, 81/111), they had received information from the websites or apps (67%, 74/111), and they had downloaded forms from the websites or apps (45%, 50/111).

Almost half of the participants had bought or ordered goods or services via the Internet (e-shopping) during the last three months (47%, 52/111), one third of the participants had never tried e-shopping (33%, 37/111). Different kinds of goods and services were ordered or bought during the last 12 months, for instance computer-based teaching materials, sports equipment, drugs/medicine, and computer equipment.

Regarding security on the Internet a bank ID or mobile bank ID was mostly used by the participants to identify themselves on the Internet (85%, 94/111). All but three participants (3%, 3/111) had identified themselves on the Internet.

With regard to the digital competence of the participants, few had participated in any formal IT education during the last 12 months. Eleven (10%, 11/111) had participated in free IT education online and two (2%, 2/111) had paid for IT education.

When asked about having a disability the majority of the participants (74%, 89/120) acknowledged they had a disability, but the most of them (86%, 77/120) considered that they were not affected by their disability when using the computer or the Internet.

**Fig. 2:**
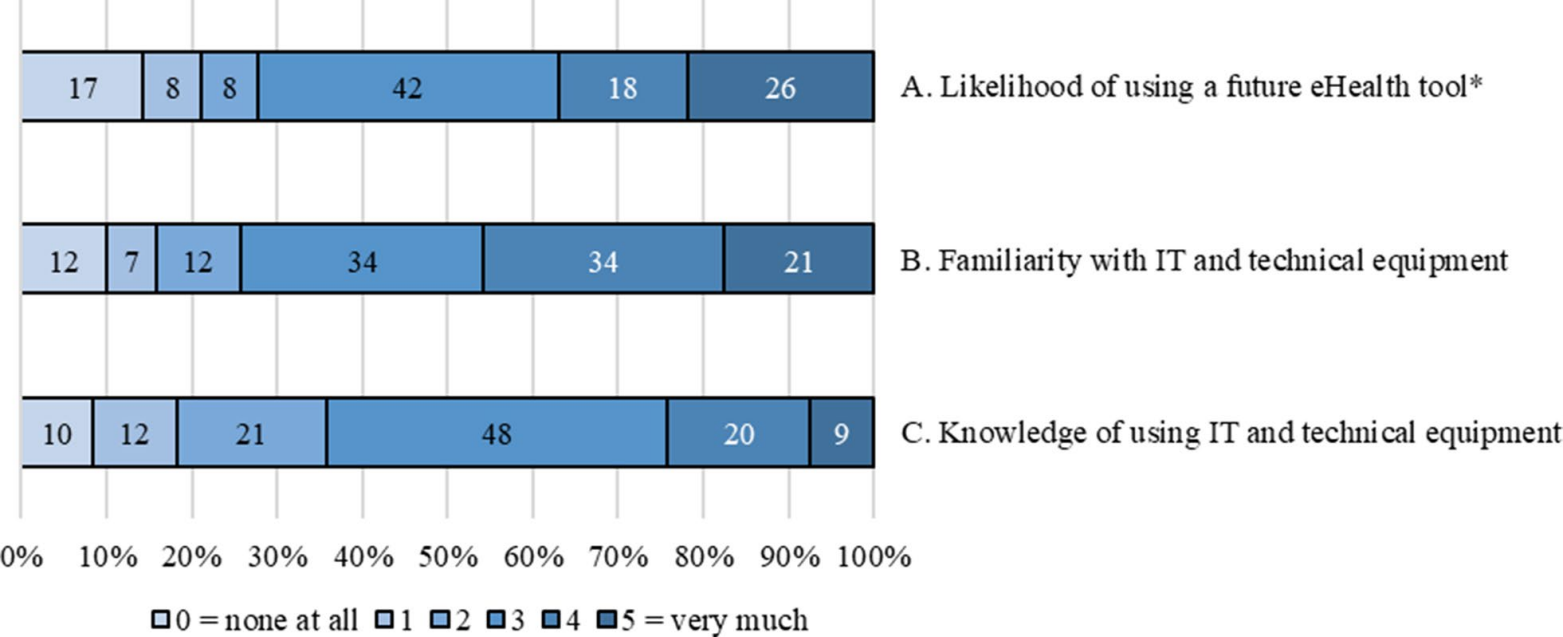
Showing 120 participants’ graded level of (A) likelihood of using a future eHealth tool designed for promoting physical activity and exercise training, (B) participants’ graded familiarity with IT and technical equipment, and (C) participants’ knowledge of using IT and technical equipment, presented in numbers. *One missing value

The self-rated question concerning the degree of probability of using a future digital eHealth tool adapted for people with COPD, showed that the participants rated their probability of using such a tool as high. Their familiarity with using IT and technical equipment was also high, as well as the grading of their knowledge of using the IT and technical equipment. See Fig. 2.

Half of the participants had used an Internet-based support for COPD. Their opinions on content, functions, and prospects in a future eHealth tool for COPD varied. The most common requirements were a tool or platform containing evidence-based and trustworthy general information on COPD (including for example medication, exercise training, inhalation and breathing techniques), a way to communicate (chat) with other people with COPD and with health care providers (physiotherapist, dietician, nurse, physician) and also individually adjusted programmes for exercise training. Getting support and help with motivation from both health care providers and others with COPD via prompts, chat rooms and/or digital information board was also raised as a wish for a future eHealth tool.

## Discussion

This is, to our knowledge, the first study to use a standardised questionnaire to investigate access to and use of the Internet in people with COPD. We found that people with COPD in Sweden had high access to the Internet and to different technical equipment related to IT. The majority were frequent users of the Internet and had sufficient knowledge to enable the use. Many also expressed a high likelihood of using a future eHealth tool for COPD, if available.

The results in this study showed that people with COPD had higher access to the Internet in the household than the general population (age 65–74) in Sweden, i.e., 92% versus 77%. Accordingly the people with COPD also had higher access to desktop Internet connections (70% vs 58%) and higher access to mobile Internet connections (62% vs 44%) [14]. Some recent reports from 2020 showed that access to the Internet and technical equipment is very high in Sweden, compared to countries in Europe and worldwide [14, 30]. There is a big difference from developing nations, shown by the United Nations (UN) where less than 20% of the population use the Internet [31]. One explanation for the high access and usage in Sweden is probably the fact that Sweden is a highly developed nation and that a majority of Swedes consider that e-services simplify things in life [32]. The government in Sweden has a national vision of eHealth for the year 2025 that highlights the opportunities offered by digitalisation and eHealth and focuses on increased independence and participation in social life. This national vision of eHealth might also be an explanation for the high access to and use of digital alternatives [33].

Changes in access to the Internet and usage of IT in recent years show that seldom/non-users of the Internet are only approximately 4% of the Swedish population, which was also confirmed in this study. Some known factors that can decrease the use of the Internet are decreased cognitive function and a high level of loneliness [30]. However, this was not assessed in the present study, and may therefore be seen as a limitation. The seldom/non-users of the Internet are often represented by older, non-working, lower educated people living alone with or without a disability [13, 30]. However, these previously known aspects of seldom/non-users of the Internet were not fully confirmed in this study; even though our sample included participants who were mostly older, non-working, some living alone, all of whom had COPD, they were still to a high extent frequent users of the Internet. Few of the participants acknowledged any impairments due to their disability (COPD and/or other), when they were using the Internet and technical equipment. That most of participants were highly educated may be an explanation for this, but may also be seen as a limitation, as we did not reach the whole span of different education levels. Only a few of the participants had had any formal IT education. Nevertheless, they felt they had sufficient digital competence and found themselves able to handle required tasks in the areas they used on the Internet. This is an important “take home message” for health care providers who need to be aware of any scepticism when considering using eHealth tools for people with COPD. This implies people with COPD could continue to be involved in and benefit from participation in digital activities, despite several known factors mentioned above speaking ill of Internet use in people with COPD.

In the present study, the majority of the individuals with COPD had used the Internet for habitual activities, many also had participated in social networking with calls via the Internet and some had booked appointments. This particular knowledge is of great importance when developing a future digital tool with the goal of reaching more people with COPD and offering a more flexible way to PR. It has also been shown that during the COVID-19 pandemic, older people in particular have begun to use the Internet and social media more than before in Sweden [13]. To reduce any digital difficulties, the design and development of digital equipment and applications must consider the needs of the least experienced [30]. Furthermore, it has been shown that a familiar device is preferable to reach higher compliance when using eHealth [17]. In the present study the participants expressed that they preferred an eHealth tool for COPD including trustworthy general information on COPD. No preferences were expressed regarding a specific device but some mentioned apps and a tool where they could get support and help. It has also been shown elsewhere that the use of health apps is increasing rapidly, which could be related to their usability, i.e. the easier they are to use, the more they increase users’ self-efficacy and willingness to use them [34]. But there is still a lack of apps containing evidence-based recommendations for PR in COPD and with the ability to individualise treatment according to a person’s current needs. Considering the future approach to COPD and the digitalisation of PR, this will be an important issue to bear in mind.

When recruiting participants for the present study, the current restrictions due to the COVID-19 pandemic as of March 2020 for people over 70 years of age and/or in risk groups to visit health care providers limited the number of participants available. In order to obtain a representative sample, the inclusion period was prolonged by 2 months, within which the target number was reached. A limitation of the present study is a selection bias since the health care providers approaching the presumptive participants did not register how many persons that declined participation. This might have affected the external validity of the results. It might also be a limitation, that the sample was collected through referrals from health care givers, which suggests participants included in the present study, already had contact with the health care and might not be representing the whole COPD population in Sweden. Since a confirmed diagnosis of COPD was required, health care providers were a suitable option for help with recruitment, and due to the GDPR there were no direct way of reaching a wider sample from the whole COPD population in Sweden. This may have led to a selection bias, where possibly the ones with the highest socio-economic standard, the most interested, the most familiar with and/or experienced in IT agreed to take part. However, the recruiting health care providers represented different socio-economic areas and were well informed of the importance of gathering presumptive participants with different experiences of IT. This also resulted in the inclusion of some participants with no experience of IT, which may be seen as a strength. There were mostly females accepting to participate in the present study which might be a concern in regard to generalizability. In Sweden, the prevalence of COPD has been shown to be slightly higher in women than in men [35].

There is a general consensus that response rates of 70% and above are required to ensure that the sample group is sufficiently representative of the target population [36]. In the present study the response rate of the group of participants who reported interest in the present study was 88%.

Another strength is that both rural areas and the centre of big cities were represented, since participants were recruited from all health care regions in Sweden. Although the majority lived in the Stockholm region, many different areas and living conditions were represented. This could reflect any possible differences in the matter of access to, and usage of the Internet and technical equipment [14]. Spirometry data for the participants COPD stadiums (I–IV) were not accessible in this study due to the general data protection regulation (GDPR). Therefore the GOLD gradings (A–D) were used [1], which could be an inconvenience when comparing our findings to other studies on COPD. Another strength is the method used with telephone interviews, which led to very few missing data. The interviewers were also able to clarify questions if the participant needed any further information.

The results of the present study indicate a high likelihood of the eHealth tools being used among people with COPD, in Sweden. We have seen that digitalisation is an important part of our society and that there is also a need for different ways to keep up with the rapid progress of digitalisation in health care. In order to reach a larger part of the COPD population, the possibility of using eHealth to a larger extent, both as a supplemental and appropriate way of communicating with participants, will be essential to delivering PR to those in need of it. Moreover, the present study was conducted in Sweden, which makes it uncertain whether these findings can be generalised to another country’s population of people with COPD. When conducting additional research these concerns should be taken in consideration.

## Conclusion

Our findings indicate that people with COPD in Sweden have high access to, and the ability to use the Internet and IT. They use IT frequently and most of them take part in the digital society and the opportunities there, and even to a higher extent than the general population of the same age. Participants expressed requests for a future eHealth tool for COPD to include evidence-based and trustworthy information on COPD, but also to include access to individually adjusted exercise training, and support, enable communication with others and with health care providers. The present study showed that people with COPD may be a suitable group for the use of digital health care. Future research on adapting an eHealth tool for people with COPD customised for their needs and demands is of great importance.

## Data Availability

The datasets generated during the current study are not publicly available, since the content of sensitive information could compromise the privacy of research participants. Datasets are available from the corresponding author on reasonable request.

## Abbreviations

app: Mobile application
CAT: The COPD assessment test
COPD: Chronic obstructive pulmonary disease
eHealth: Electronic health
GDPR: The general data protection regulation
GOLD: Global initiative for chronic obstructive lung disease
ICT: Information and communication technologies
IT: Information technology
PR: Pulmonary rehabilitation
